# Association of physical activity trajectories over 8 years and risk of knee replacement: data from the osteoarthritis initiative

**DOI:** 10.1186/s12891-024-07710-9

**Published:** 2024-07-26

**Authors:** Yining Wang, Ziyuan Shen, Xing Xing, Liru Ge, Faming Pan, Guoqi Cai

**Affiliations:** 1 Department of Epidemiology and Biostatistics, School of Public Health, Anhui Medical University, Hefei, Anhui 230032 China; 2 The Inflammation and Immune-Mediated Diseases Laboratory of Anhui Province, Anhui Medical University, 81 Meishan Road, Hefei, Anhui 230032 China; 3 Menzies Institute for Medical Research, University of Tasmania, Hobart, TAS 7000 Australia

**Keywords:** Knee replacement, Osteoarthritis, Physical activity, Trajectory

## Abstract

**Background:**

To identify physical activity (PA) trajectories in adults with or at risk of knee osteoarthritis and to evaluate the association of PA trajectories with incident knee replacement (KR).

**Methods:**

This study used data from the Osteoarthritis Initiative. The Physical Activity Scale for the Elderly and the KR were assessed annually from baseline to 9 years. Individuals were included if they did not undergo KR surgery at baseline and had data on PA at ≥ 1 visit before KR. Latent class growth mixture Modeling was used to identify the optimal trajectories of PA before KR. Log-binomial regression models were used to assess the association between PA trajectories and the risk of KR. Data analyses were conducted in all individuals and those with radiographic osteoarthritis (ROA) and significant knee pain (Western Ontario and McMaster Osteoarthritis Index pain score of ≥ 5 on a 0–20 scale) at baseline, respectively.

**Results:**

Of 4731 participants (mean age 61.1 years, 58.5% female), four distinct and slightly declined PA trajectories were identified. Compared to individuals with a “Low” PA trajectory, those with “Medium-low”, “Medium-high”, or “High” PA trajectories were not significantly associated with the risk of KR (risk ratios: 0.97–1.19, all *p* > 0.05). Similar PA trajectories and associations with the risk of KR were observed in the subgroups of individuals with radiographic osteoarthritis and those with significant knee pain at baseline, respectively.

**Conclusion:**

In participants with or at risk of knee osteoarthritis, PA slightly declines over time and may play no role in the risk of KR.

**Supplementary Information:**

The online version contains supplementary material available at 10.1186/s12891-024-07710-9.

## Introduction

The incidence and prevalence of knee osteoarthritis (KOA) have increased markedly with the rising obesity rate and aging of the population [1, 2]. In large weight-bearing joints (e.g. knee and hip), osteoarthritis (OA) is a whole joint disease involving all joint tissues (cartilage, meniscus, subchondral bone, infrapatellar fat pad, and synovial membrane) [3]. OA causes an enormous burden to the population and often leads to disability requiring surgical intervention [2] such as knee replacement (KR). Kirsten et al. found that about 7.1% of newly diagnosed OA patients would undergo KR over 2.6 years [4], and more than 95% of KR is due to knee OA [5]. With the rising prevalence of KOA, the number of KR has gradually increased [6, 7]. While KR is the only option and is highly effective for end-stage KOA, KR recipients can experience persistent pain and long-term complications [5, 8, 9].

Physical activity (PA) can reduce the risk of adverse health conditions such as falling [10], cardiovascular disease (CVD), and mortality [11, 12]. It is generally believed that PA is also beneficial for KOA [13, 14], and guidelines have consistently and strongly recommended PA and exercise for patients with KOA [15, 16]. However, a meta-analysis indicates that recreational PA is not related to the risk and progression of radiographic KOA and knee pain [17], and another study using MRI to assess the progression of KOA has even shown that ≥ 10,000 steps/day may be detrimental to knee structural changes such as bone marrow lesions (BMLs) and cartilage defects [18]. Meanwhile, previous studies have also shown the self-reported PA in the highest quartile did not affect the risk of developing OA [19] and may have a beneficial effect on knee articular cartilage [20]. In a systematic review of randomized controlled trials, high-intensity exercise showed no clinically important benefits for pain and function compared with low-intensity exercise programs [21]. Moreover, while PA generally declines with age, and KOA is disabling [22, 23], studies across different populations have consistently shown that PA levels in KOA patients are comparable to those without KOA or knee pain [24–26]. Previous studies have found that patients with or at risk for KOA have distinct PA trajectories, and the distinct PA patterns may have different effects on the progression of KOA [26, 27]. As an important outcome of KOA, it is unclear whether the risk of KR varies among adults with different PA trajectories. We hypothesized that high PA trajectories may be protective against the risk of KR. Therefore, the aims of this study were (1) to identify PA trajectories in adults with or at risk of KOA, and (2) to evaluate the association of PA trajectories with incident KR.

## Materials and methods

### Study sample

**Fig. 1:**
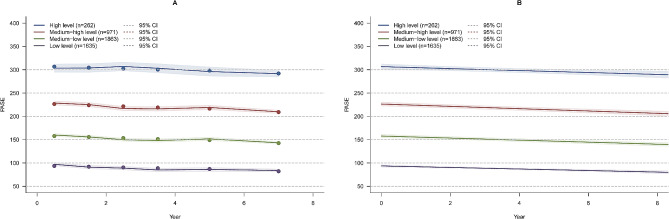
Study flowchart

This study was conducted in accordance with the Strengthening the Reporting of Observational Studies in Epidemiology (STROBE) guidelines [28]. The data used in this study were derived from the Osteoarthritis Initiative (OAI), a multicenter, longitudinal, prospective observational study of participants with or at an increased risk of KOA. The risk factors for inclusion were older age (> 45 years), frequent knee symptoms, regular use of medications for knee symptoms, being overweight, a history of knee injury or surgery, a family history of OA, the presence of Heberden’s nodes, and engaging in activities that involve repetitive knee bending [29]. The OAI cohort included 4,796 participants aged–45–79 years at the time of recruitment. Ethics approval was obtained from the institutional review boards of the four clinical centers (Memorial Hospital of Rhode Island, the Ohio State University, the University of Pittsburgh, and the University of Maryland/Johns Hopkins) that recruited OAI participants. All the participants provided written informed consent. A detailed description of the OAI study is available at https://nda.nih.gov/oai/about-oai.html. Data of the OAI from 0 to 96 months were used in this study, where month 0 was considered as the baseline. A total of 4731 participants who did not have knee replacement surgery before baseline and had data on PA at ≥ 1 visit before a KR were included in the study; two subgroups were identified: knee pain and radiographic OA (ROA) at baseline (Fig. 1).

### Assessment of PA

PA was assessed annually from baseline to 8 years using the Physical Activity Scale for the Elderly (PASE) questionnaire [30, 31]. The PASE covers 3 domains of activity: leisure activities, household activities, and occupational activities. The period covered by PASE is the past 7 days. The frequency, duration, and intensity of activity over the past seven days were recorded, and the total PASE score was calculated, which ranged from 0 to 400 or more (the maximum score was 556 in this population, with higher scores indicating greater PA. For each participant, data on PASE used for the evaluation of PA trajectories were restricted to visits before any KR was conducted.

### Assessment of incident KR

Facts and dates of KR were self-reported and adjudicated from radiographs or medical records at the baseline and each follow-up visit. An outcome event was considered when KR surgery was performed during follow-up.

### Assessment of knee pain and radiographic OA

Knee pain was assessed using the Western Ontario and McMaster Osteoarthritis Index (WOMAC) pain subscale over the past seven days [32]. Each item of the WOMAC pain subscale was evaluated using a five-point Likert scale (0 none, 1 mild, 2 moderate, 3 severe, 4 extreme), and the knee pain score was the sum of the individual item scores (0–20), with a higher number representing worse symptoms. Participants were considered to have significant knee pain at baseline if the WOMAC pain score was ≥ 5 [33, 34].

Knee radiographs were obtained using a fixed flexion knee radiograph, and the Kellgren-Lawrence (KL) grade was evaluated. Two projects in the OAI (Projects 15 and 37/42) were conducted to measure the KL grade, with two OAI experts blinded to each other’s reading and all other data. We used the ‘worst’ KL grade from the two projects to merge the duplicated data, as recommended in the OAI handbook. Participants were considered to have ROA at baseline if the KL grade of any knee was ≥ 2 [35].

### Covariates

Covariates were selected based on previous studies [24, 25, 36]. We used baseline data on age, sex (male, female), body mass index (BMI, kg/m^2^), race (white, black, other), marital status (married or similar, widowed, divorced, separated, never married), level of education (< high school, high school graduate, some college, college graduate, some graduate school, and graduate degree) and income, history of knee injury (yes/no) and knee surgery (yes/no), use of medications (non-steroidal anti-inflammatory drugs [NSAIDs], steroids, painkillers and acetaminophen), physical function (SF-12 physical score), KL grade (the worse KL grade in both knees ), WOMAC pain score (the worse score in both knees). To account for the potential impact of seasonality on PA, we further adjusted for the month of the year that evaluated PA at baseline.

### Statistical analysis

First, we estimated Latent Class Growth Mixture Modeling (LCGMM) using the lcmm package, a mixed model for multivariate longitudinal outcomes using a maximum likelihood estimation method, to reveal the latent class and predicted trajectories of PA [37]. They can be used to identify latent subgroups, classes, or clusters of individuals based on their common growth trajectories over time. The GRoLTS-checklist was used to ensure that these analyses are reproducible [38]. Individuals who completed at least one PASE measurement were included in the analysis. To determine the optimal number of trajectories, we tested models with different numbers and forms (linear, quadratic, and cubic) of the potential trajectories [26, 39]. The final number of trajectories was established according to the following criteria: (a) Akaike information criterion (AIC) [40], Bayesian information criterion (BIC) [41], and sample size adjusted Bayesian information criterion (SABIC) [42], with smaller values indicating a better model fit; (b) entropy (range from 0 to 1), with higher values indicating a better class separation; (c) each growth trajectory was assigned at least 5% of the total population; (d) the mean posterior probability of each trajectory class was greater than 70% [43]; (e) the interpretability and research significance of the identified trajectories. We established PA trajectories for all participants, participants with knee pain at baseline, and participants with ROA at baseline.

Second, we evaluated the associations of PA trajectories with the risk of KR using log-binomial regression models, and the results are shown as risk ratios (RR) with 95% confidence intervals (CI). Adjustment was made for age, sex, BMI, race, level of education and income, knee injury, knee surgery, use of medications, physical function, KL grade, and WOMAC pain scores. If the log-binomial regression models failed to converge, the RR was estimated using Poisson distribution and robust standard errors (SEs) [44]. The interactions between PA trajectories and BMI or sex at baseline were assessed. The results are reported as RRs and 95% CI. These analyses were also conducted in participants with significant knee pain (WOMAC pain score ≥ 5) and ROA at baseline as sensitivity analyses. We conducted another sensitivity analysis restricting participants with data on PA at 2 or more visits.

Missing data on covariates were addressed using multiple imputations with chained equations (MICE) [45]. Ten imputations were conducted using complete covariates and non-missing values of the predictor and outcome measures at baseline, assuming random missing values.

All analyses were performed using R, version 4.2.3 [46]. A two-sided p-value less than 0.05 was considered statistically significant.

## Results

### Participants

Of the 4731 participants included in this study, 2766 (58%) were female, the mean (standard deviation) age was 61.05 (9.17) years, 1468 (31%) had significant knee pain (i.e. WOMAC pain score ≥ 5), and 2512 (53.1%) had ROA at baseline. Table 1 shows the baseline characteristics of the participants. The time point and the number of KR can be seen in Supplementary Table 5.

**Table 1:** Baseline characteristics of study participants with different trajectories of physical activity

characteristics	total	low level	medium-low level	medium-high level	high level	
	*N* = 4731	*N* = 1635	*N* = 1863	*N* = 971	*N* = 262	p
Age, year, mean (SD)	61.05 (9.2)	65.11 (8.8)	61.3 (8.7)	55.84 (7.3)	53.28 (5.5)	< 0.001
Body mass index, kg/m^2^, mean (SD)	28.6 (4.8)	28.96 (4.9)	28.42 (4.8)	28.34 (4.7)	28.64 (5)	0.005
Gender (%)						
Male	1965 (41.5%)	544 (27.7%)	751 (38.2%)	495 (25.2%)	175 (8.9%)	
Females	2766 (58.5%)	1091 (39.4%)	1112 (40.2%)	476 (17.2%)	87 (3.1%)	< 0.001
Race (%)						
White or Caucasian	3737 (79.1%)	1227 (32.8%)	1500 (40.1%)	801 (21.4%)	209 (5.6%)	
Black or African American	862 (18.2%)	360 (41.8%)	316 (36.7%)	141 (16.4%)	45 (5.2%)	
Other	127 (2.7%)	47 (37%)	43 (33.9%)	29 (22.8%)	8 (6.3%)	< 0.001
Education level (%)						
Less than high school	164 (3.5%)	101 (61.6%)	45 (27.4%)	13 (7.9%)	5 (3%)	
High school	599 (12.8%)	267 (44.6%)	228 (38.1%)	84 (14%)	20 (3.3%)	
Some college	1125 (24%)	375 (33.3%)	462 (41.1%)	230 (20.4%)	58 (5.2%)	
College graduate	990 (21.1%)	313 (31.6%)	397 (40.1%)	225 (22.7%)	55 (5.6%)	
Some graduate school	392 (8.4%)	112 (28.6%)	165 (42.1%)	90 (23%)	25 (6.4%)	
Graduate degree (Master or PhD)	1420 (30.3%)	454 (32%)	548 (38.6%)	320 (22.5%)	98 (6.9%)	< 0.001
Income						
< 10k	159 (3.6%)	92 (57.9%)	41 (25.8%)	25 (15.7%)	1 (0.6%)	
10-25k	444 (10.2%)	221 (49.8%)	157 (35.4%)	49 (11%)	17 (3.8%)	
25-50k	1113 (25.5%)	449 (40.3%)	438 (39.4%)	197 (17.7%)	29 (2.6%)	
50-100k	1592 (36.4%)	482 (30.3%)	662 (41.6%)	349 (21.9%)	99 (6.2%)	
> 100k	1065 (24.4%)	256 (24%)	416 (39.1%)	291 (27.3%)	102 (9.6%)	< 0.001
Marital						
married or similar	3130 (66.7%)	998 (31.9%)	1258 (40.2%)	683 (21.8%)	191 (6.1%)	
widowed	373 (8%)	186 (49.9%)	148 (39.7%)	35 (9.4%)	4 (1.1%)	
divorced	676 (14.4%)	241 (35.7%)	261 (38.6%)	143 (21.2%)	31 (4.6%)	
separated	85 (1.8%)	32 (37.6%)	31 (36.5%)	15 (17.6%)	7 (8.2%)	
never married	426 (9.1%)	164 (38.5%)	148 (34.7%)	87 (20.4%)	27 (6.3%)	< 0.001
Medications						
painkill	118	61 (51.7%)	35 (29.7%)	18 (15.3%)	4 (3.4%)	0.001
steroids	91	43 (47.3%)	34 (37.4%)	12 (13.2%)	2 (2.2%)	0.033
acetaminophen	509	235 (46.2%)	184 (36.1%)	71 (13.9%)	19 (3.7%)	< 0.001
NSAIDs	343	147 (42.9%)	133 (38.8%)	48 (14%)	15 (4.4%)	0.001
knee injury	1939	595 (30.7%)	744 (38.4%)	453 (23.4%)	147 (7.6%)	< 0.001
knee surgery	1020	295 (28.9%)	385 (37.7%)	261 (25.6%)	79 (7.7%)	< 0.001
Radiographic osteoarthritis (%)^e^	2512 (56.5%)	942 (37.5%)	464 (18.5%)	989 (39.4%)	117 (4.7%)	< 0.001
WOMAC, mean (SD)^f^	3.48 (3.7)	3.87 (4)	3.34 (3.6)	3.16 (3.6)	3.16 (3.6)	< 0.001

a: All information was collected at baseline

b: SD: standard deviation

c: Values are the number (%) unless indicated otherwise

d: NSAIDs = nonsteroidal anti-inflammatory drugs

e: At least one knee with radiographic osteoarthritis (KL-grade ≥ 2)

f: WOMAC pain score ≥ 5 (5–20)

### PA trajectories

**Fig. 2:**
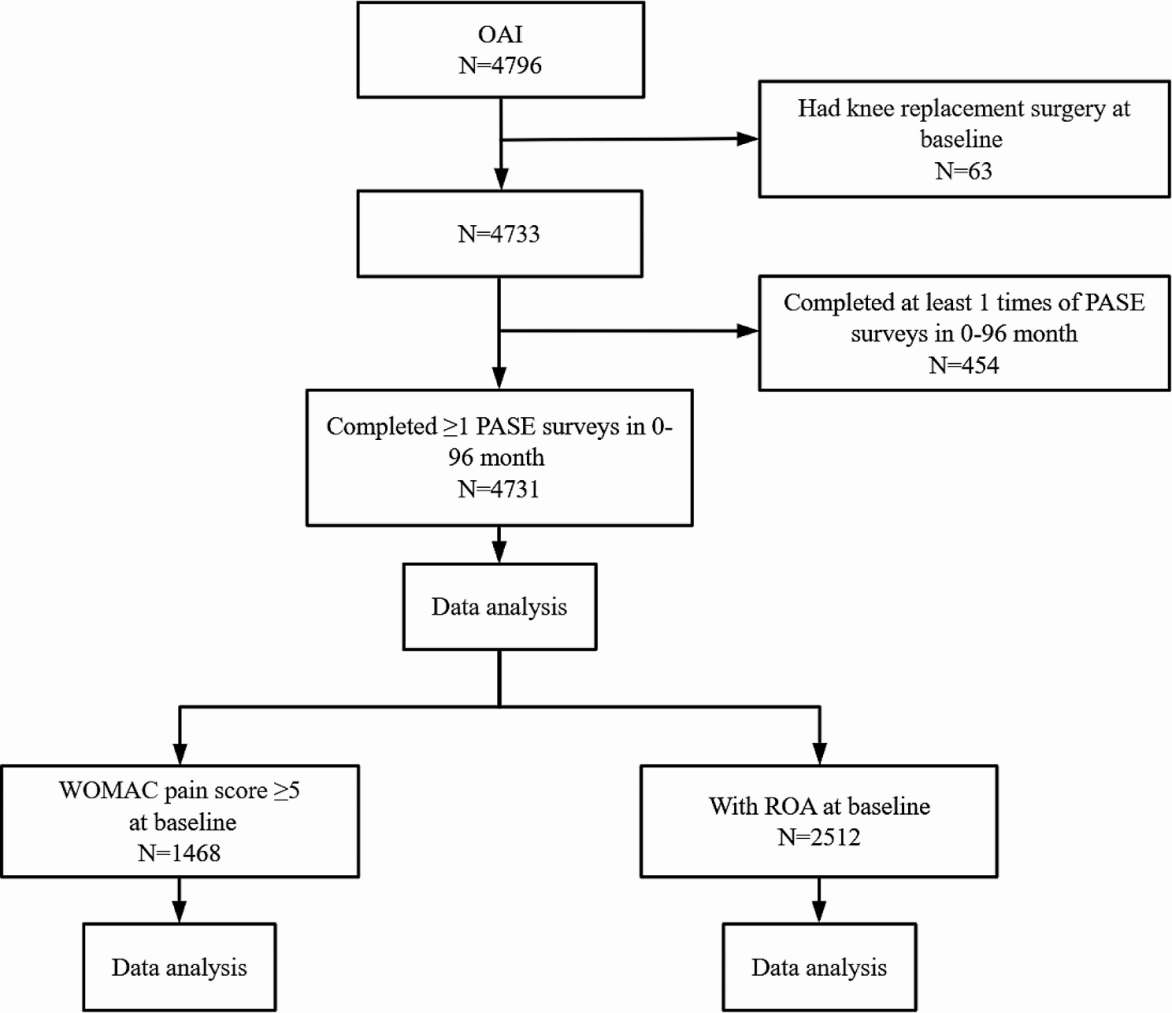
Estimated and observed mean trajectory in total participants (Fig. 2**A**) and the predicted mean trajectories in total participants (Fig. 2**B**). Solid lines show class-specific mean predicted levels as a function of 8 years estimated from the best fitting growth mixture model (4-class linear latent class growth mixture modeling), shaded areas indicate estimated 95% confidence intervals

Participants were separated into four different latent classes, estimated and observed mean trajectories of PA Fig. 2A, and the predicted mean growth curves of the four distinct trajectories are shown in Fig. 2B. These were identified and labeled as “Low level” (*n* = 1635, 34.56%), “Medium-low level” (*n* = 1863, 39.38%), “Medium-high level” (*n* = 971, 20.52%), “High level” (*n* = 262, 5.54%). PA trajectories in participants with significant knee pain or ROA were similar (Supplementary Fig. 1). Compared to participants with Low levels of PA, those who had Medium-low, Medium-high, or High-level PA were more likely to be younger and have higher income and education levels (Table 1). The four trajectories had a high mean posterior probability, all higher than 0.8, indicating a good model fit (Supplementary Table 1). Similarly, trajectory analyses also showed a good model fit in participants with significant knee pain and those with ROA at baseline (Supplementary Tables 2 and Supplementary Table 3). The characteristics of PASE scores over time are reported in Supplementary Table 4.

### Association of PA trajectories with risk of KR

Among the 4731 participants, 417 underwent KR during the 9-year follow-up period. Compared to the “Low level” PA trajectory, individuals with a trajectory of “Medium-low level” PA (adjusted RR, 1.15, 95% CI, 0.91–1.57), “Medium-high level” PA (adjusted RR, 1.07, 95% CI, 0.73–1.59), or “High level” PA (adjusted RR, 0.97, 95% CI, 0.51–1.84) were not associated with the risk of KR (Table 2).

**Table 2:** Association between physical activity trajectories and knee replacement

	Crude model			Adjusted model^a^	
	RR^b^	P		RR (95% CI^c^)	P
**Total** (*n* = 4731)					
Low level	1.00 (ref)			1.00 (ref)	
Medium-low level	0.97 (0.79 to 1.18)	0.74		1.19 (0.91 to 1.57)	0.21
Medium-high level	**0.72 (0.55 to 0.95)**	**0.02**		1.07 (0.73 to 1.59)	0.73
High level	**0.59 (0.35 to 0.98)**	**0.04**		0.97 (0.51 to 1.84)	0.92
**Baseline with pain**^d^(*n* = 1468)					
Low level	1.00 (ref)			1.00 (ref)	
Moderate level	0.98 (0.77 to 1.26)	0.89		1.07 (0.84 to 1.36)	0.59
High level	**0.60 (0.39 to 0.92)**	**0.02**		0.81 (0.53 to 1.25)	0.34
**Baseline with ROA**^e^(*n* = 2512)					
Low level	1.00 (ref)			1.00 (ref)	
Moderate level	0.86 (0.71 to 1.04)	0.13		0.96 (0.79 to 1.17)	0.72
High level	**0.71 (0.51 to 0.98)**	**0.04**		0.83 (0.59 to 1.15)	0.26

^a^ Adjusted for sex, age, body mass index, race, education, marital, income, Kellgren-Lawrence grade, WOMAC pain score, the use of medications, history of knee injury and knee surgery, sf12 physical score

^b^ Risk ratio

^c^ Confidence interval

^d^ WOMAC pain score ≥ 5 (5–20) at baseline

^e^ At least one knee with radiographic osteoarthritis (KL-grade ≥ 2) at baseline

### Sensitivity analysis

Missing data on covariates ranged from 0.02–6.99% (Supplementary Table 6). Complete case analyses did not materially alter the main findings (Supplementary Table 7). PA trajectories in participants with significant knee pain or ROA were similar, and there were 3 distinct PA trajectories for both subgroups: “Low-level”, “Medium level”, and High level” (Supplementary Fig. 1). No significant associations were found between PA trajectories and KR risk in participants with significant knee pain or ROA (Table 2). In participants with 2 or more PA assessments, PA trajectories and their association with the risk of KR were similar to the main findings (Supplementary Fig. 2 and Supplementary Table 8).

## Discussion

In middle-aged and older adults with or at an increased risk of KOA, four distinct slightly declined PA trajectories (High, Medium-high, Medium-low, and Low) were identified over the 8-year follow-up period, but PA trajectories may play no role in the risk of KR. Similar PA trajectories and association with the risk of KR were also observed in subgroups of participants with significant knee pain and those with ROA at baseline. These results suggest that PA trajectories are similar in adults with or at risk for KOA and that High-level PA does not increase the risk of progression to end-stage KOA.

PA levels within the four trajectories only slightly declined during the eight years, indicating that substantial changes in the average level of PA were rare over a long period in participants with or at an increased risk of KOA. We found that most (> 70%) participants showed a Low- or Medium-low level PA trajectory and that more (80%) participants with ROA and significant knee pain at baseline showed a Low- or Moderate-level PA trajectory. This is consistent with the finding that, among the general population, approximately 80% of adults and adolescents in the US are insufficiently active [47]. This may be due to many reasons, such as pain, catastrophizing, and injury [48]. A previous study showed that when patients catastrophize pain in the morning, they would engage in less PA and more sedentary behavior throughout the day [48].

Several studies have investigated the association between PA and the risk of KR, with inconsistent findings. In a meta-analysis that combined three case-control studies, the odds of KR in runners were lower than those in the controls [49]. In contrast, Wang et al. used data from a large population-based cohort study showing that PA, especially vigorous activity, increased the risk of KR [50]. Munugoda et al. also found that every 1000 steps/day increase in ambulatory activity, as measured by pedometers, was associated with a 9% greater risk of KR [51]. Nonetheless, Skou et al. indicated that PA, assessed by the PASE score, was not significantly associated with the risk of KR with knee pain at both short-term (2–2.5 years) and long-term (7 years) follow-ups [52]. However, these studies focused on the association between PA at one time point and the risk of KR, which failed to capture the dynamic characteristics of PA. The present study identified the PA trajectories of participants and explored their association with the risk of KR. We found that a Higher-level PA trajectory was not associated with a statistically significant incident KR, irrespective of whether the participants had ROA or significant knee pain at baseline. This study evaluated PA using PASE, which cannot well distinguish between activities that involve heavy, repeated load of the joints and those that do not, thus we were not able to evaluate the type of activity on the risk of TKR in this study. However, a previous study has summarized relevant evidence showing that the risk of KR is increased in those who conduct repetitive high-impact sports like soccer, team handball, and ice hockey, but not normal exercises like jogging, gymnastics, and swimming.

PA is essential for the management of KOA by controlling weight and strengthening muscles, thereby reducing the burden on the joints and the risk of knee injury or pain. Most patients consider KR surgery when they have worse radiographic KOA, serious chronic knee pain, stiffness, and functional impairment that significantly impair their quality of life. Strong evidence indicates that increased levels of PA can decrease knee pain in adults with OA [53]. Doré et al. in an MRI study showed a detrimental effect of high-intensity PA on cartilage loss [18], but this effect may be transient and cannot overweigh the beneficial effect of PA [54]. In the present study, individuals with higher PA levels did not have significantly increased odds of incident KR, even after adjusting for the ability to perform PA. The Physical Activity Guidelines for Americans indicate that both aerobic and muscle-strengthening PA are beneficial, and moving more and sitting less will benefit nearly everyone [47]. PA counseling should act as part of the standard care for individuals at high risk of KOA, especially at an early stage when PA engagement is more attainable.

The strengths of this study include the large sample size with long-term follow-up and the use of at least one PASE visit to avoid uncertain estimates in latent classes. Our study had several limitations. First, PA was assessed using a self-reported PASE questionnaire, which could be subject to reporting bias. Accelerometers can provide a more precise estimation of PA assessment but would be costly, time-consuming, and burdensome to participants. Second, assessments of PA were implemented annually, which may have missed some variations in these subjective measures. Third, as this was an observational study, residual confounding cannot be excluded. However, we included multiple confounders, especially those that may influence the implementation of KR, based on the previous literature. Fourth, PASE cannot well distinguish between activities that involve heavy, repeated load of the joints and those that do not, thus we were not able to evaluate the type of activity on the risk of TKR in this study.

In conclusion, among 4731 participants with or at high risk of KOA, four distinct and slightly declined PA trajectories were identified, with more than 80% of participants having low- or medium-level PA over 8 years. Participants with different PA levels had a similar risk of KR.

### Electronic supplementary material

Below is the link to the electronic supplementary material.

Supplementary Material 1

## Data Availability

The datasets generated and/or analyzed during the current study are available from the corresponding author upon reasonable request.

## Abbreviations

AIC: Akaike information criterion
BIC: Bayesian information criterion
BMI: Body mass index
BMLs: Bone marrow lesions
CI: Confidence intervals
CVD: Cardiovascular disease
KL: Kellgren-Lawrence
KR: Knee replacement
LCGMM: Latent class growth mixture Modeling
NSAIDs: Non-steroidal anti-inflammatory drugs
OAI: Osteoarthritis Initiative
PA: Physical activity
PASE: Physical Activity Scale for the Elderly
PP: Posterior probability
ROA: Radiographic osteoarthritis
RR: Risk ratios
SABIC: Size-adjusted Bayesian information criterion
SEs: Standard errors
STROBE: Strengthening the Reporting of Observational Studies in Epidemiology
WOMAC: Western Ontario and McMaster Osteoarthritis Index
