# Supplementary material for: Association of physical activity trajectories over 8 years and risk of knee replacement: data from the osteoarthritis initiative
Source: BMC Musculoskelet Disord. 2024 Jul 26;25:586. doi: 10.1186/s12891-024-07710-9 (PMC11282720; doi:10.1186/s12891-024-07710-9)
Supplement: Supplementary file 1 — Supplementary Material 1 [file 12891_2024_7710_MOESM1_ESM.docx]

| degree | G | loglik | conv | npm | AIC | BIC | SABIC | entropy | Class membership (%) | Mean posterior probabilities (%) | prob>0.7 |
| --- | --- | --- | --- | --- | --- | --- | --- | --- | --- | --- | --- |
| linear | 1 | -156050 | 1 | 3 | 312106 | 312125.4 | 312115.9 | 1 | 1 | 1 | 1 |
|  | 2 | -151473.1 | 1 | 6 | 302958.1 | 302996.9 | 302977.8 | 0.8356791 | 67.41/32.59 | 96.09/93.35 | 95.36/91.50 |
|  | 3 | -150174.6 | 1 | 9 | 300367.2 | 300425.4 | 300396.8 | 0.7914583 | 46.57/39.93/13.51 | 91.5/87.84/91.91 | 89.15/82.58/89.36 |
|  | **4** | **-149772.5** | **1** | **12** | **299568.9** | **299646.5** | **299608.4** | **0.7550028** | **34.56/39.38/20.52/5.54** | **87.95/85.45/82.14/91.52** | **84.71/79.20/73.81/89.31** |
|  | 5 | -149622.6 | 1 | 15 | 299275.2 | 299372.1 | 299324.4 | 0.7256586 | 26.55/36.19/24.41/10.86/1.99 | 83.2/77.76/80.29/84.99/89.73 | 75.56/67.64/71.17/80.93/86.17 |
| quadratic | 1 | -156048.2 | 1 | 4 | 312104.4 | 312130.3 | 312117.6 | 1 | 1 | 1 | 1 |
|  | 2 | -151469.3 | 1 | 8 | 302954.5 | 303006.2 | 302980.8 | 0.8357363 | 67.41/32.59 | 96.11/93.34 | 95.20/91.50 |
|  | 3 | -150167.8 | 1 | 12 | 300359.7 | 300437.2 | 300399.1 | 0.7916015 | 46.63/39.91/13.46 | 91.49/87.85/92.01 | 89.26/82.52/89.48 |
|  | 4 | -149764.8 | 1 | 16 | 299561.7 | 299665 | 299614.2 | 0.7547639 | 34.56/39.4/20.5/5.54 | 87.96/82.05/85.48/91.7 | 84.59/74.25/79.28/89.31 |
|  | 5 | -149639.4 | 1 | 20 | 299318.9 | 299448.1 | 299384.6 | 0.7309295 | 32.97/39.19/11.03/10.84/5.96 | 87.13/80.84/75.97/70.4/90.7 | 82.56/73.68/62.45/49.12/86.88 |
| cubic | 1 | -156043.6 | 1 | 5 | 312097.2 | 312129.5 | 312113.6 | 1 | 1 | 1 | 1 |
|  | 2 | -151461.9 | 1 | 10 | 302943.8 | 303008.5 | 302976.7 | 0.8359211 | 67.45/32.55 | 96.07/93.41 | 95.30/91.69 |
|  | 3 | -150156.5 | 1 | 15 | 300343 | 300439.9 | 300392.3 | 0.792595 | 46.82/39.89/13.3 | 91.54/87.86/92.23 | 89.16/82.41/90.30 |
|  | 4 | -149752.3 | 1 | 20 | 299544.6 | 299673.8 | 299610.2 | 0.7550165 | 34.52/20.63/39.34/5.52 | 88.01/85.36/82.09/92.09 | 84.69/79.10/74.48/90.04 |
|  | 5 | -149621 | 1 | 25 | 299292 | 299453.5 | 299374.1 | 0.7302506 | 32.76/11.18/10.93/39.12/6 | 87.03/75.09/71.35/80.72/91.31 | 82.39/60.68/50.48/72.99/87.68 |

# Supplementary Table 1: Fit statistics of latent class mixed model for all participants:

G: number of latent classes; loglik: log-likelihood of the model; conv status of convergence: =1 if the convergence criteria were satisfied, =2 if the maximum number of iterations was reached; AIC: Akaike information criterion; BIC: Bayesian information criterion; SABIC: sample size adjusted Bayesian information criterion; prob: table of posterior classification and posterior individual class-membership probabilities.

| degree | G | loglik | conv | npm | AIC | BIC | SABIC | entropy | Class membership (%) | Mean posterior probabilities (%) | prob>0.7 |
| --- | --- | --- | --- | --- | --- | --- | --- | --- | --- | --- | --- |
| linear | 1 | -43826.24 | 1 | 3 | 87658.49 | 87674.36 | 87664.83 | 1 | 1 | 1 | 1 |
|  | 2 | -42558.03 | 1 | 6 | 85128.05 | 85159.8 | 85140.74 | 0.8206739 | 69.28/30.72 | 95.57/92.57 | 94.30/89.80 |
|  | **3** | **-42230.51** | **1** | **9** | **84479.01** | **84526.64** | **84498.05** | **0.7508723** | **43.19/41.49/15.33** | **89.54/85.27/91.21** | **86.75/78.82/87.11** |
|  | 4 | -42104.64 | 1 | 12 | 84233.28 | 84296.78 | 84258.66 | 0.7498526 | 37.06/40.53/18.26/4.16 | 87.49/81.61/86.36/88.76 | 83.64/72.10/80.60/85.25 |
|  | 5 | -42063.22 | 1 | 15 | 84156.45 | 84235.82 | 84188.17 | 0.7304344 | 35.9/10.42/8.99/40.46/4.22 | 87.42/73.58/74.04/79.79/87.97 | 84.25/55.56/56.82/68.18/82.26 |
| quadratic | 1 | -43825.66 | 1 | 4 | 87659.32 | 87680.49 | 87667.78 | 1 | 1 | 1 | 1 |
|  | 2 | -42556.69 | 1 | 8 | 85129.39 | 85171.72 | 85146.31 | 0.8214352 | 69.35/30.65 | 95.58/92.6 | 94.40/90.22 |
|  | 3 | -42228.79 | 1 | 12 | 84481.57 | 84545.07 | 84506.95 | 0.7511463 | 43.6/41.21/15.19 | 89.28/85.46/91.45 | 86.09/79.17/86.10 |
|  | 4 | -42098.23 | 1 | 16 | 84228.46 | 84313.13 | 84262.3 | 0.7501546 | 36.99/18.19/40.67/4.16 | 87.59/86.58/81.55/89.2 | 84.53/81.65/72.19/85.25 |
|  | 5 | -42056.34 | 1 | 20 | 84152.68 | 84258.51 | 84194.98 | 0.733837 | 36.24/10.08/40.46/8.72/4.5 | 87.43/75.37/80.17/73.12/85.01 | 83.83/58.78/68.52/53.91/77.27 |
| cubic | 1 | -43825.55 | 1 | 5 | 87661.1 | 87687.55 | 87671.67 | 1 | 1 | 1 | 1 |
|  | 2 | -42556.03 | 1 | 10 | 85132.06 | 85184.98 | 85153.21 | 0.821599 | 69.35/30.65 | 95.65/92.49 | 94.40/89.78 |
|  | 3 | -42226.99 | 1 | 15 | 84483.98 | 84563.36 | 84515.71 | 0.7507741 | 43.46/41.14/15.4 | 89.31/85.52/91.08 | 85.89/79.97/86.28 |
|  | 4 | -42095.23 | 1 | 20 | 84230.46 | 84336.29 | 84272.76 | 0.7501803 | 36.99/18.46/40.33/4.22 | 87.52/86.04/81.83/89.5 | 83.98/80.07/72.97/82.26 |
|  | 5 | -10000000 | 4 | 25 | 2000000050 | 2000000182 | 2000000103 | 1 | 0/0/0/0/0 | NaN/NaN/NaN/NaN/NaN | NaN/NaN/NaN/NaN/NaN |

# Supplementary Table 2: Fit statistics of latent class mixed model for participants with significant knee pain at baseline:

G: number of latent classes; loglik: log-likelihood of the model; conv status of convergence: =1 if the convergence criteria were satisfied, =2 if the maximum number of iterations was reached; AIC: Akaike information criterion; BIC: Bayesian information criterion; SABIC: sample size adjusted Bayesian information criterion; prob: table of posterior classification and posterior individual class-membership probabilities.

# Supplementary Table 3: Fit statistics of latent class mixed model for participants with ROA at baseline:

| degree | G | loglik | conv | npm | AIC | BIC | SABIC | entropy | Class membership (%) | Mean posterior probabilities (%) | prob>0.7 |
| --- | --- | --- | --- | --- | --- | --- | --- | --- | --- | --- | --- |
| linear | 1 | -83784.7 | 1 | 3 | 167575 | 167593 | 167583 | 1 | 1 | 1 | 1 |
|  | 2 | -81290.5 | 1 | 6 | 162593 | 162628 | 162609 | 0.8451 | 68.43/31.57 | 96.36/93.3 | 95.35/91.68 |
|  | **3** | **-80542.1** | **1** | **9** | **161102** | **161155** | **161126** | **0.81185** | **47.09/40.37/12.54** | **92.66/89.05/93.1** | **90.96/86.19/90.79** |
|  | 4 | -80313.7 | 1 | 12 | 160651 | 160721 | 160683 | 0.77871 | 39.85/37.1/18.43/4.62 | 89.27/83.67/86.2/92.92 | 78.22/87.02/83.37/87.93 |
|  | 5 | -80189.7 | 1 | 15 | 160409 | 160497 | 160449 | 0.75026 | 26.63/10.39/24.72/37.02/1.23 | 84.39/89/82.48/79.56/91.13 | 77.88/87.36/75.36/71.18/83.87 |
| quadratic | 1 | -83782.5 | 1 | 4 | 167573 | 167596 | 167584 | 1 | 1 | 1 | 1 |
|  | 2 | -81287.5 | 1 | 8 | 162591 | 162638 | 162612 | 0.84526 | 68.35/31.65 | 96.36/93.32 | 95.46/91.45 |
|  | 3 | -80538.3 | 1 | 12 | 161101 | 161171 | 161132 | 0.81202 | 47.21/40.25/12.54 | 92.58/89.15/92.94 | 90.81/86.45/90.79 |
|  | 4 | -80310 | 1 | 16 | 160652 | 160745 | 160694 | 0.77889 | 37.14/39.89/18.39/4.58 | 89.56/83.6/86.99/91.51 | 87.35/77.45/83.33/88.70 |
|  | 5 | -80238.3 | 1 | 20 | 160517 | 160633 | 160570 | 0.75741 | 34.99/8.16/39.81/12.22/4.82 | 89.01/75.47/82.51/75.15/92.42 | 84.76/62.44/76.6/60.91/90.08 |
| cubic | 1 | -83780.8 | 1 | 5 | 167572 | 167601 | 167585 | 1 | 1 | 1 | 1 |
|  | 2 | -81285.5 | 1 | 10 | 162591 | 162649 | 162617 | 0.84516 | 68.31/31.69 | 96.29/93.46 | 95.45/91.46 |
|  | 3 | -80534.9 | 1 | 15 | 161100 | 161187 | 161140 | 0.81244 | 47.25/40.33/12.42 | 92.63/89.04/92.93 | 90.82/85.98/91.99 |
|  | 4 | -80303.1 | 1 | 20 | 160646 | 160763 | 160699 | 0.7766 | 36.9/39.41/19.03/4.66 | 83.69/89.46/87/91.15 | 86.73/78.59/80.75/94.02 |
|  | 5 | -80180.1 | 1 | 25 | 160410 | 160556 | 160477 | 0.75247 | 26.95/10.15/24.76/36.98/1.15 | 83.93/0.8923/82.58/79.29/90.94 | 78.73/87.45/75.08/71.80/86.21 |

G: number of latent classes; loglik: log-likelihood of the model; conv status of convergence: =1 if the convergence criteria were satisfied, =2 if the maximum number of iterations was reached; AIC: Akaike information criterion; BIC: Bayesian information criterion; SABIC: sample size adjusted Bayesian information criterion; prob: table of posterior classification and posterior individual class-membership probabiliti

# Supplementary Table 4: Characteristics of PASE score over time

| Month |  | Total | |  | Baseline with pain | |  | Baseline with ROA | |
| --- | --- | --- | --- | --- | --- | --- | --- | --- | --- |
|  |  | N | Mean (sd) |  | N | Mean (sd) |  | N | Mean (sd) |
| 0 |  | 4704 | 161.29 (82.6) |  | 1457 | 155.2 (84.8) |  | 2496 | 156.82 (81.4) |
| 12 |  | 4258 | 157.48 (82.1) |  | 1245 | 153.09 (85.5) |  | 2363 | 151.93 (80.9) |
| 24 |  | 3982 | 153.66 (80.8) |  | 1128 | 149.02 (83.8) |  | 2209 | 149.41 (79.6) |
| 36 |  | 3833 | 151.54 (81.8) |  | 1062 | 147.98 (84.9) |  | 2096 | 144.63 (79.2) |
| 48 |  | 3631 | 154.67 (82.3) |  | 986 | 147.32 (83.1) |  | 1957 | 147.92 (80.8) |
| 72 |  | 3277 | 152.53 (82.6) |  | 841 | 145.71 (85.0) |  | 1714 | 145.14 (80.3) |
| 96 |  | 3107 | 147.97 (80.8) |  | 766 | 143.51 (84.2) |  | 1596 | 141.58 (80.2) |

SD: standard deviation

| Month | Total | Baseline with pain | Baseline with ROA |
| --- | --- | --- | --- |
|  | number | number | number |
| 12 | 23 | 16 | 18 |
| 24 | 38 | 23 | 38 |
| 36 | 51 | 33 | 50 |
| 48 | 51 | 30 | 50 |
| 60 | 54 | 29 | 52 |
| 72 | 48 | 26 | 47 |
| 84 | 51 | 27 | 46 |
| 96 | 57 | 29 | 51 |
| 108 | 44 | 20 | 35 |

# Supplementary Table 5: Characteristics of KR over time

# Supplementary Table 6: Baseline covariates missing information

|  | Total (%) | baseline with pain (%) | baseline with ROA (%) |
| --- | --- | --- | --- |
| BMI | 0.09 | 0.24 | 0.17 |
| Education | 0.77 | 1.20 | 0.76 |
| Race | 0.09 | **0.16** | **0.04** |
| Marital | 0.77 | 1.12 | 0.76 |
| Income | **6.99** | **8.16** | **7.25** |
| Acetophenone | 0.19 | 0.32 | 0.21 |
| Painkiller | 0.09 | 0.24 | 0.17 |
| steroids | 0.16 | 0.40 | 0.21 |
| NSAIDs | 0.23 | 0.40 | 0.25 |
| Knee Injury | 0.68 | 0.80 | 0.89 |
| Knee Surgery | 0.12 | 0.24 | 0.08 |
| Radiographic osteoarthritis (%) | 0.93 | 1.52 | 0.00 |
| WOMAC | **0.02** | 0.00 | 0.00 |

# Supplementary Table 7: Association between physical activity trajectories and risk of knee replacement in complete case analysis

|  | Adjusted model^[[1]](#footnote-1)^ | |
| --- | --- | --- |
|  | RR^[[2]](#footnote-2)^ (95%CI^[[3]](#footnote-3)^) | P |
| **Total (n=4041)** |  |  |
| Low level | 1.00 (ref) |  |
| Medium-low level | 1.16 (0.91 to 1.64) | 0.19 |
| Medium-high level | 1.12 (0.76 to 1.77) | 0.49 |
| High level | 1.43 (0.51 to 2.12) | 0.91 |
| **Baseline with pain^[[4]](#footnote-4)^ (n=1187)** |  |  |
| Low level | 1.00 (ref) |  |
| Moderate level | 1.03 (0.80 to 1.32) | 0.87 |
| High level | 0.86 (0.55 to 1.33) | 0.56 |
| **Baseline with ROA^[[5]](#footnote-5)^ (n=2272)** |  |  |
| Low level | 1.00 (ref) |  |
| Moderate level | 0.99 (0.81 to 1.22) | 0.96 |
| High level | 0.86 (0.60 to 1.22) | 0.39 |

|  | Crude model |  |  | Adjusted model |  |
| --- | --- | --- | --- | --- | --- |
|  | RR (95%CI) | P |  | RR (95%CI) | P |
| **Total** |  |  |  |  |  |
| Low level | 1.00 (ref) |  |  | 1.00 (ref) |  |
| Medium-low level | 0.97 (0.77 to 1.23) | 0.81 |  | 1.24 (0.92 to 1.67) | 0.17 |
| Medium-high level | 0.73 (0.54 to 0.99) | 0.04 |  | 1.18 (0.79 to 1.76) | 0.42 |
| High level | 0.60 (0.33 to 1.01) | 0.07 |  | 1.09 (0.53 to 2.15) | 0.80 |

# Supplementary Table 8: Association between physical activity trajectories and risk of knee replacement in complete case analysis in total participants excluding individuals with only 1 visit.

Adjusted for sex, age, body mass index, race, education, marital, income, Kellgren-Lawrence grade, WOMAC pain score, the use of medications, history of knee injury and knee surgery, sf12 physical score, the month baseline visited;

b participants excluding individuals with only 1 visit.

#
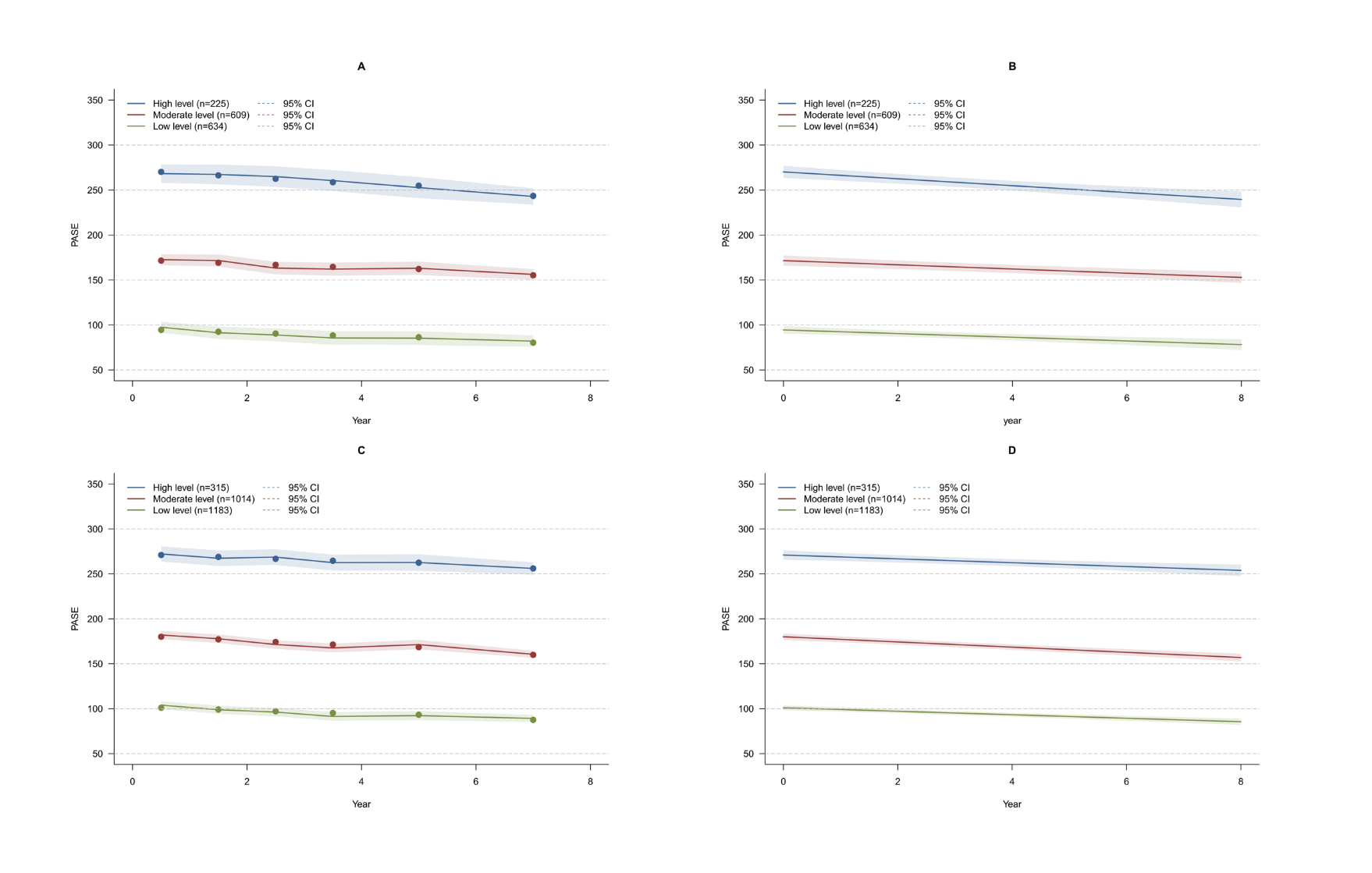
Supplementary Figure 1

Estimated and observed mean trajectory for baseline with pain (**Supplementary Figure 1A**) and baseline with radiographic osteoarthritis (**Supplementary Figure 1C**) and the predicted mean trajectories for baseline with pain (**Supplementary Figure 1B**) and baseline with radiographic osteoarthritis (**Supplementary Figure 1D**).

Solid lines show class-specific mean predicted levels as a function of 8 years estimated from the best fitting growth mixture model (3-class linear latent class growth mixture modeling), shaded areas indicate estimated 95% confidence intervals

# Supplementary Figure 2


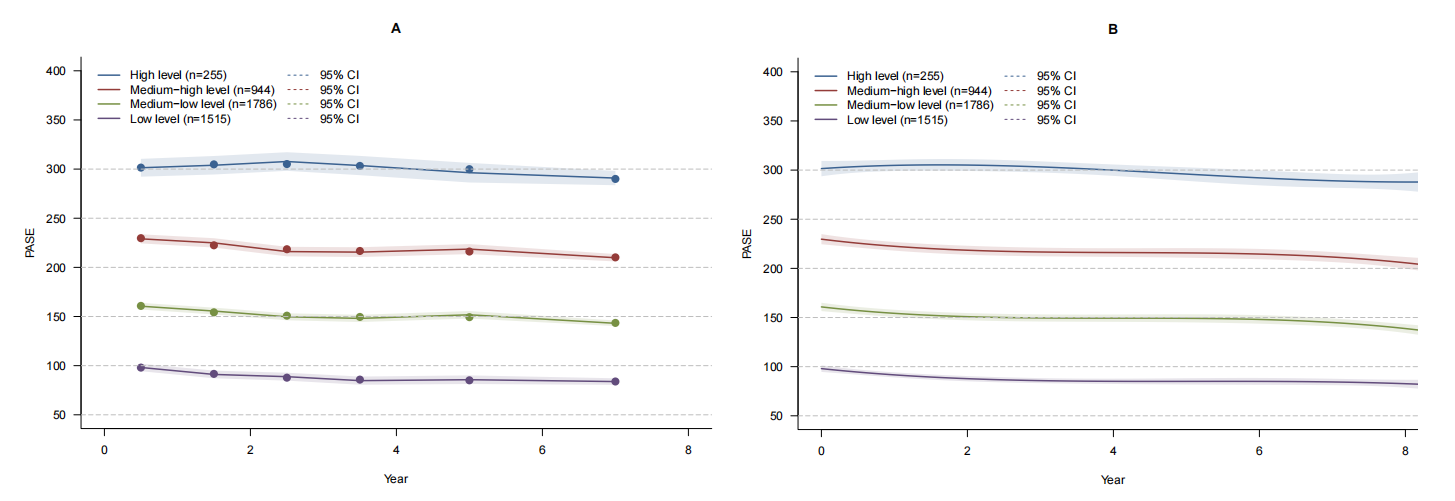


Estimated and observed mean trajectory in total participants (**Supplementary Figure 2A**) and the predicted mean trajectories in total participants (**Supplementary Figure 2B**) excluding individuals with only 1 visit. Solid lines show class-specific mean predicted levels as a function of 8 years estimated from the best fitting growth mixture model (4-class cubic latent class growth mixture modeling), shaded areas indicate estimated 95% confidence intervals

1. Adjusted for sex, age, body mass index, race, education, marital, income, Kellgren-Lawrence grade, WOMAC pain score, the use of medications, history of knee injury and knee surgery, sf12 physical score, the month baseline visited; [↑](#footnote-ref-1)
2. Risk ratio; [↑](#footnote-ref-2)
3. confidence interval; [↑](#footnote-ref-3)
4. WOMAC pain score≥5 (5-20) at baseline; [↑](#footnote-ref-4)
5. At least one knee with radiographic osteoarthritis (KL-grade≥2) at baseline. [↑](#footnote-ref-5)
